# Application of a peer learning and assessment model in an undergraduate pharmacy course

**DOI:** 10.1186/s12909-023-04352-8

**Published:** 2023-05-22

**Authors:** Liyuan Yang, Yi Wang

**Affiliations:** School of Medicine and Pharmacy, Laboratory for Marine Drugs and Bioproducts of Qingdao National Laboratory for Marine Science and Technology, Ocean University of China, Qingdao, 266003 China

**Keywords:** Pharmacy education in university, Peer assessment, Peer learning, Feedback efficiency, Learning ability

## Abstract

**Background:**

Timely and accurate feedback is a crucial component for effective undergraduate learning. However, with the expansion of university enrolment in China, student numbers have increased rapidly and, in traditional university classrooms, it is often difficult for the teacher – as the only evaluator – to accommodate students’ diverse needs and learning styles, and provide timely learning feedback. In our teaching practice research, we combined mutual peer evaluation with cooperative learning, and proposed a peer learning and assessment model (PLAM) that encouraged students to cooperate and compete, leading to greater efficiency in giving feedback. The ultimate goal was to improve students’ learning ability. This study aimed to investigate the effect and influencing factors of PLAM in an undergraduate course entitled ‘Medicinal Chemistry of Natural Products’.

**Methods:**

We surveyed the entire pharmacy student body (95 students). Each student was required to provide feedback to the other members within the same study group and students in other groups. We evaluated the effectiveness of PLAM in five aspects: basic information, learning attitude, participation, interpersonal relationship, and organizational approach. The questionnaire was administered online using the Star survey platform. Data were exported to Excel and meta-analysis was performed using SPSS.

**Results:**

PLAM effectively increased feedback efficiency, enhancing students’ learning interest and ability. An ordered logistic regression analysis model was used to analyze the factors influencing the PLAM learning effect. Three factors – learning attitude, participation, and interpersonal relationship – explained up to 71.3% of the model.

**Conclusions:**

The PLAM adopted in this research is an effective learning and evaluation model that can promote collaborative learning and increase learning enthusiasm. It is more suitable for knowledge expansion learning and comprehensive practical learning where teachers cannot be present for the entire process. Students should be encouraged to establish appropriate learning attitudes and a positive group atmosphere. PLAM can positively impact college curriculum learning and could be extended to other teaching domains.

**Supplementary Information:**

The online version contains supplementary material available at 10.1186/s12909-023-04352-8.

## Background

Traditional Chinese-style university classrooms face many challenges, including outdated teaching methods and content, limited teacher-student interaction, passive students, inefficient assessments, and the need to compromise in group situations [1]. Teaching evaluations typically focus on results while ignoring the process of learning. The knowledge acquired by the end of the course is considered most important, while the process of knowledge transmission in the teaching-learning environment is typically ignored [2].

With increasing Chinese university enrolment and subsequent growth in student numbers, teachers struggle to accommodate students’ diverse needs and learning styles, and give timely learning feedback. There is a need for classroom teaching to be transformed and the feedback process from teachers to students improved. Evaluation is the main motivation for students and an important means to measure the degree and quality of student learning [3]. Combining the learning process with feedback evaluation is key to student learning. As university teaching moves towards more flexible methods, online and blended learning, student-driven learning, and student participation, many teachers now use peer assessment to evaluate students. In this approach, feedback is provided by both teachers and students. Peer assessment can be used as an evaluation method to complement teacher evaluation and improve learning outcomes [4–7]. It can reduce the pressure teachers face to feedback on students’ learning, and students can accurately and consistently judge their peers’ performance [8].

## Introduction to peer assessment

Peer assessment is also called peer evaluation or peer feedback. It relies on peer relationships and the interaction between students, and refers to a feedback process in which one or more evaluators (a single student or group) provide scores or descriptive evaluations of the output of one or more evaluation objects [9]. Peer assessment is not a new teaching method. It has a long history in basic and university education that dates back to the 1970s [10]. Since then, global research on peer assessment has increased significantly [11], but to date it has rarely been used in Chinese university teaching. Instead, Chinese universities tend to rely on traditional evaluation methods, with teachers typically the only evaluators of students’ learning. This approach requires updating for more effective and evidence-based classroom practice.

Peer assessment addresses the limitations of traditional classrooms and provides a new approach to education. It guides students to actively participate in the evaluation of their peers, thereby increasing the effectiveness of learning and feedback [12].Assessment is considered to comprise two main types, each with a distinct purpose. Summative assessment is used for certification or grading purposes, and formative assessment is used for learning and development. Peer assessment is a type of formative evaluation that aims to promote learning to build understanding, and can stimulate acquisition of higher-level skills, such as responsibility-sharing, reflection, discussion, and cooperation [13–15].

In many peer assessment studies, the core activity is providing feedback and obtaining feedback from others to improve the performance of each group member and/or the entire group. Peer assessment benefits all students – those who are evaluated and the evaluators themselves [16]. Many researchers believe that peer assessment has great potential to support self-assessment, self-regulation, and autonomous learning [17–19]. Studies have shown that peer assessment can promote students’ cognitive development, evaluation and critical abilities, metacognitive awareness and social emotional development [20–23].

Previous peer assessment studies have focused on the positive impact of evaluation on students. Students themselves are the main agents of learning, and through the process of peer assessment, they express their ideas, share thoughts with others, accept constructive feedback from peers, and jointly construct knowledge through dialogue and interaction [24]. However, peer assessment has shortcomings, including the lack of emphasis on teamwork [25] – students may become independent and competitive with each other. Effective peer assessment can be time-consuming to administer using traditional methods [25, 26], especially in overcrowded classrooms, and can place a significant burden on students if each student is required to evaluate other students. Moreover, some students tend to comment only superficially and give suggestions that are not helpful for revision [27]. Research shows that students find peer assessment stressful and uncomfortable [28, 29]. Therefore, some researchers are skeptical about peer assessment.

Peer assessment can greatly improve feedback efficiency, but has shortcomings for cooperative learning. To address the current model’s shortcomings and improve the quality of teaching, we modified the traditional peer assessment approach, combining peer assessment and peer learning. We proposed a peer learning and assessment model (PLAM) that helps students compete and cooperate, thereby improving feedback efficiency. The ultimate goal was to improve students’ learning ability.

## Peer learning and assessment model (PLAM)

Literature indicates that peer assessment has many benefits for teaching practice, but also some shortcomings. In particular, there is a lack of emotional construction with a collaborative basis. It is also time consuming, reducing the efficiency of learning and evaluation. In our teaching practice, we set out to adjust the peer assessment process and introduce peer learning to address these challenges.

Peer learning is a two-way reciprocal learning activity [30], a learning model in which students cooperate, learn from each other, think and solve problems together under the teacher’s guidance. It is more effectively applied to knowledge-intensive courses [31]. The advantages of peer learning include practical and emotional support from peers, increased self-confidence and cooperation, and reduced anxiety [32–37]. Peer learning emphasizes cooperation rather than competition, and respects the experiences and backgrounds of participants [38].

Therefore, we combined the advantages of peer learning and peer assessment, and proposed a peer learning assessment model (PLAM) comprising the two core processes of peer learning and assessment. Peer learning involves students helping each other learn content and collaborate to complete tasks [39]. Peer assessment is a process in which peers give and receive feedback from each other. Therefore, PLAM moves from a one-way process led by teachers to a constructive and dynamic process of cooperative learning and student-to-student interaction [40].

We started by training students on how PLAM works and how to provide evaluations. Our classes were divided into multiple study groups. The grades, styles, and characteristics of the students in each group differed. Each group cooperated to complete a learning task and improve their teamwork when learning. Mutual evaluations between groups occurred to promote competition in the classroom. At the same time, the students in each group commented on the contributions of their peers, creating competition within the group. Therefore, each student only needed to evaluate other members of the same group and other groups, reducing the burden and saving time. Teachers gave guidance at appropriate times. Thus, the peer-to-peer mutual evaluation model relied on student evaluation, and teacher evaluation supplemented the process evaluation model that improved the efficiency of learning evaluation. There was no teacher supervision or feedback during this time because cooperative group learning is a process outside of the classroom. Therefore, the teacher’s role in PLAM is to guide students in giving evaluations, and provide authoritative comments on the final outcome of group work to correct any errors arising from group learning.

## Objective

Traditional Chinese medicine has played an important role in clinical treatment in China, and ‘Medicinal Chemistry of Natural Products’ – the subject underpinning Chinese medicine – is a course that every pharmacy student must master because it is fundamental for the growth of medicinal knowledge and professional skills. The Medicinal Chemistry of Natural Products course also contains many trivial knowledge points. Students need to have a good knowledge of organic chemistry, analytical chemistry, and spectral analysis, and the ability to apply databases and scientific and technological software to analyses. Further, outdated textbook content is always included in the course, and advanced knowledge of such material must be acquired. Therefore, we divided the class into study groups, and each group worked together to complete an introduction to knowledge beyond the textbook using PLAM. The purpose of this research was to analyze the effects of PLAM on the teaching of this undergraduate chemistry course, consider factors influencing PLAM, and provide references enabling modification of the teaching model in follow-up studies.

## Research questions

Does PLAM promote efficient student access to feedback? Does PLAM promote improvement of students’ learning abilities in group work? What aspects affect student evaluation of this model?

In the Methods section that follows, we provide details of the PLAM implementation and the content and methodology of the questionnaire. The Results section presents our data analysis and description of the main findings together with findings from the adjusted model. Finally, further improvements of PLAM are discussed in the Discussion section.

## Methods

### Teaching practice and data collection

**Fig. 1:**
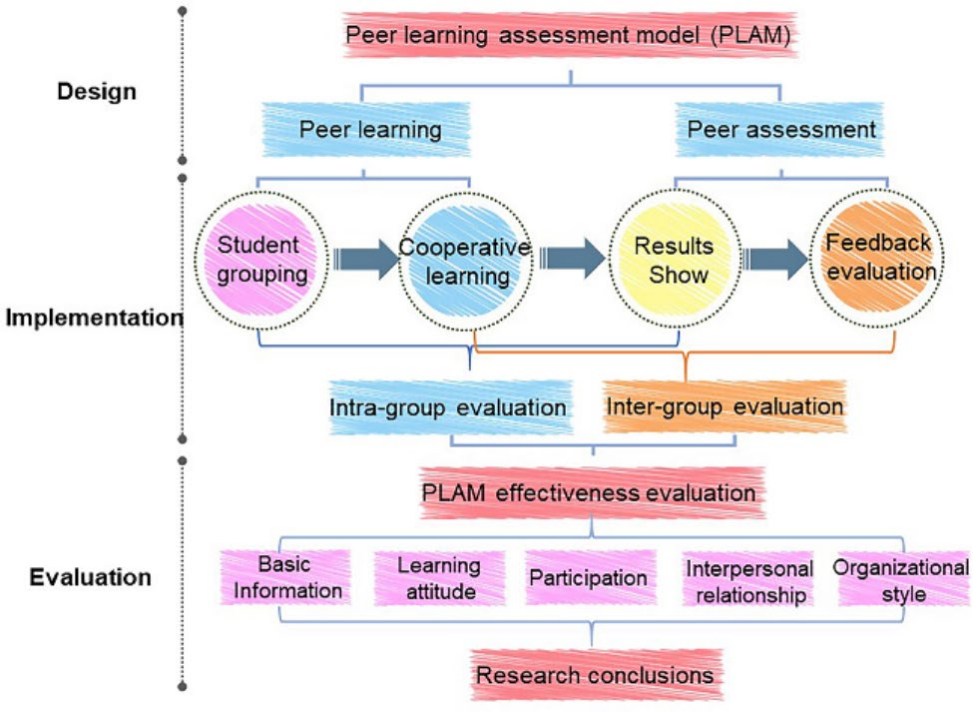
Schematic diagram of the peer learning and assessment model (PLAM)

The study analyzed the effects of PLAM and factors influencing it in an undergraduate chemistry course. A total of 95 students participated. ‘Medicinal Chemistry of Natural Products’ is a professional course offered by the School of Medicine at Ocean University of China for third-year undergraduates. The learning approach follows the traditional face-to-face teaching method, aided by online Blackboard teaching and an integrated platform to enable homework submission. Teaching time for this course is 32 contact hours, divided into two parallel classes, with 47 students in one class and 48 in the other. As shown in Fig. 1, PLAM was divided into three phases: design, implementation, and evaluation. In the design phase, students freely formed themselves into ten study groups, each comprising 8–11 people. Group members were required to collaborate on a review. The content required a search for natural medicine or drugs inspired by natural products around a type of bioactivity, e.g., the introduction and expansion of anti-malarial compounds, such as artemisinin. During this process of peer learning, members needed to collaborate to determine the division of labor, grading criteria, slide production, and presentation of results. Each student was required to provide feedback and grading to the other members of the group and to the other groups mid-way through and at the end of the session – the peer evaluation process. In the implementation stage, intra- and inter-group evaluations were intertwined, forming the process evaluation.

At the end of the course and before final exams, data collection questionnaires were distributed. This timing was to ensure that students had participated in PLAM throughout the course and were capable of giving their evaluations of PLAM. The students were informed that the purpose of the questionnaire was to collect data for research into teaching. We evaluated the effectiveness of PLAM in five dimensions: basic information, learning attitude, participation, interpersonal relationship, and organizational approach.

### Questionnaire

After a preliminary literature review, Schunn’s scale for modification was selected for use in this study. Schunn’s scale measures the influence of student attitude, interpersonal factors and organizational style on participation in online mutual peer evaluation [41–43]. We divided the questionnaire into three parts, with 41 questions in total. The first part focused on basic characteristics of the learner, including gender, grade point ranking during the undergraduate period, learning style, and peer learning experience. The second section considered factors influencing PLAM, such as learning attitude, participation, interpersonal relationships, and organizational method. The third was the evaluation of the effect of the peer learning evaluation model by learners. The second and third parts of the questionnaire used a five-point Likert scale, with options set to strongly agree (5), agree (4), neutral (3), disagree (2), and strongly disagree (1). The questionnaire used in this study is presented in the supporting information.

Based on the results of the first study, we adjusted the number of group members (reduced to 5–7) and provided guidance on group division of labor so that each student had a specific task. In a follow-up survey, we used the same questionnaire again with two classes of students (94 students in total) participating in ‘Medicinal Chemistry of Natural Products’.

### Data analysis

The questionnaire was administered online using the star survey platform (https://www.wjx.cn/). Data were exported to Excel, and SPSS (v.20, Armonk, US) was used to determine the reliability and validity of the questionnaire, and for exploratory factor analysis, frequency analysis of categorical variables, one-way ANOVA, correlation analysis, multiple linear regression analysis and one-sample *t*-tests.

### Word frequency analysis

We conducted a supplementary survey of 97 students to investigate their perceptions of PLAM. To fit with our teaching practice, we recruited students in the semester following the main study. For the survey, we selected 32 descriptive terms, both positive and negative, from the literature related to peer learning and peer assessment. Students were asked to consider their innermost feelings, regardless of right or wrong, and to choose the 10 words that best reflected their feelings.

## Results

### Reliability and validity of the scale and frequency analysis of categorical variables

We conducted reliability and sanitization tests on Schunn’s scale to determine its reliability and validity, and ensure the quality of the questionnaire because we had modified that scale to suit our situation. A total of 95 questionnaires were collected, and 36 scale questions were examined for reliability. The results are shown in Table 1. The standardized Cronbach coefficients for the five dimensions of learning attitude, participation, interpersonal relationship, organizational style, and PLAM effect were all greater than 0.7, indicating good reliability. Cronbach’s alpha based on standardized terms suggested that one item should be deleted for duplication. The standardization coefficient of the 35 items in the total dataset was 0.943, and the value range of the reliability coefficient was between 0 and 1. Scores closer to 1 indicate greater reliability, indicating the reliability of the instrument is very high.

**Table 1:** Scale reliability analysis

Dimensions	Cronbach’s Alpha	Cronbach’s alpha based on standardized terms	Number of items
Learning attitude	0.910	0.914	5
Participation	0.880	0.884	7
Interpersonal relationship	0.640	0.704	5
Organizational style	0.709	0.711	8
Model effect evaluation	0.951	0.951	10
Total scale	0.939	0.943	35

The 35-item scale was tested for structural validity. Results of exploratory factor analysis (shown in Table 2) indicated the coefficient of the Kaiser-Meyer-Olkin (KMO) test was 0.849. The coefficient of the test ranges from 0 to 1, with results closer to 1 indicating better validity. The sphericity test indicated the significance of this test was infinitely close to 0, rejecting the null hypothesis. Thus, the questionnaire has good validity. Basic information about students was collected and classified, and frequency analysis was undertaken. The statistical results are shown in Table 3.

**Table 2:** Kaiser-Meyer-Olkin (KMO) and Bartlett’s test

Kaiser-Meyer-Olkin measure of sampling adequacy		0.849
Bartlett’s sphericity test	Approximate chi-square	2625.715
	*df*	595
	Sig.	0.000

**Table 3:** Categorical variable frequency analysis

Variable	Options	Frequency	Percentage	Mean	Standard deviation
Gender	Wen	29	30.5%	1.86	0.497
	Women	66	69.5%		
Performance range	The top 20%	18	18.9%	2.85	1.288
	20–40%	21	22.1%		
	40–60%	24	25.3%		
	60–80%	21	22.1%		
	Last 20%	11	11.6%		
Self-study method	Self-study independently	67	70.5%	2.19	1.179
	Self-study together	19	20.0%		
	Basically no self-study	9	9.5%		
Self-study frequency	Once a week	22	23.2%	2.17	0.781
	2–3 times a week	35	36.8%		
	4 times a week and above	38	40.0%		
Self-study approach	Tablet	19	20.0%	1.86	0.497
	Textbooks and handouts	70	73.7%		
	Other	6	6.3%		
Have you participated in peer assessment?	Yes	86	90.5%	1.09	0.294
	No	9	9.5%		
Whether student served as team leader	Yes	20	21.1%	1.79	0.410
	No	75	78.9%		
Learning style	Divergent	35	36.8%	2.19	1.179
	Assimilator	30	31.6%		
	Gatherer	7	7.4%		
	Adaptor	23	24.2%		

### Difference analysis

The independent sample *t*-test and one-way ANOVA were used to study the differences in variables in different dimensions according to their mathematical characteristics. One-way ANOVA (see Table S1 of the Supporting Information) showed no significant difference in the performance interval of the five dimensions because the *p*-values all exceeded 0.05. However, the significance of participation was 0.066, which is close to 0.05. Although there was no significant difference, the results of multiple comparisons explain some trends. Degree of participation was linked to students’ grades. Students with the highest grades (the top 40% of students) are more active in learning than those with the lowest 20% of grades. Thus, stronger students participated more actively than lower-performing students under the peer learning evaluation model. This may be why they get good results, and the only way to gain more from PLAM is to actively participate in it. Students with scores of 60–80% aimed to improve their grades by being more enthusiastic and taking the course seriously. Their participation rate was also greater than that of weaker students. Students with the lowest grades (bottom 20%) demonstrated poor learning initiative and enthusiasm, needing more attention and guidance in future teaching-learning activities. In conclusion, PLAM is able to motivate most students to participate in learning.

The one-way ANOVA results in Table S2 show that among the five dimensions, only model effect evaluation and participation degree differ significantly in self-study methods, with *p*-values of 0.044 and 0.039, respectively. Multiple comparisons show that the model effect evaluation is related to self-study. Independent self-study occurs less than self-study with partners. Thus, students who study together communicate and cooperate more with others in daily self-study. Being adaptable when using the peer learning model leads to greater benefits from the approach. In terms of participation, independent self-study occurs less than self-study with partners because students who study independently tend to think on their own and solve problems independently. Compared with students who study with partners, they lack the experience of communicating and cooperating with others. Based on this model, participation was also low. This is partly a reflection of the potential application of PLAM. Active participation in PLAM-based teaching and learning activities will strengthen students’ ability to cooperate and communicate, and good cooperation skills are one of the most important competencies that need to be developed throughout the university education process.

Results of the one-way ANOVA in Table S3 show that of the five dimensions, only learning attitude differed significantly from the frequency of self-study. Multiple comparisons indicate that learning attitude is linked to the frequency of self-study. Students who have too many self-study times a week pay more attention to themselves than students who have a good grasp of knowledge, only a moderate number of self-study sessions a week, and are more flexible in their learning. In addition to mastering knowledge, the moderate self-study students also actively innovated. Therefore, their attitude toward peer learning was better than those who study 4 times a week or more. However, the independent sample *t*-test and one-way ANOVA showed no significant statistical differences in gender, whether students had participated in peer evaluation, whether they had served as group leaders, their approach to studying after class, and learning style in all dimensions. Thus, the null hypothesis could not be rejected because there was no significant difference in the five variables in each dimension (see Tables S4–S8 of the Supporting Information).

Based on the results of the difference test, we can conclude that PLAM can be widely applied to many different groups of students, including those with low levels of participation. Teachers need to encourage low-achieving students to actively participate in learning. They should also develop students’ cooperation and communication skills, and ensure that students have moderate levels of knowledge to benefit from the learning outcomes of PLAM.

### Correlation analysis and multiple linear regression analysis

Logistic regression is widely applied to describe and test hypotheses about the relationship between a categorical outcome variable and one or more categorical or continuous predictor variables. We investigated the relationship between the evaluation of model effects and the four dimensions, and whether there is a correlation between the four variables. We first constructed a model for correlation and regression analysis [44, 45] that can be represented as:

$$ y=f({\omega }_{1},{\omega }_{2},{\omega }_{3},{\omega }_{4})+\mu $$

where *y* is the average value of model effect evaluation, *ω*_*1*_ *~ ω*_*4*_ are the influencing factors of four dimensions, and *µ* is the error term or random disturbance term. The model effect evaluation is an ordinal categorical variable, written in logistic regression analysis as:

$$ \text{log}\left(\frac{{\pi }}{1-{\pi }}\right)={\beta }_{0}+{\beta }_{i}{X}_{i}$$

where *π* represents the probability of evaluating the effect of the model, *β*_*0*_ is a constant term, and *β*_*i*_ is the regression coefficient of different independent variables *X*_*i*_.

We used multiple linear regression analysis with learning attitude, participation, interpersonal relationship, and organizational style as independent variables, and model effect evaluation as the dependent variable. SPSS (v. 20.0) was used for correlation analysis of the model. The results showed that the three dimensions of learning attitude, engagement, and interpersonal relationships were significantly correlated at the 99% level, indicating that these three dimensions can influence each other (Table S9 in the Supporting Information). The analysis showed that the regression model has significant statistical significance (*F* = 59.434, *P* < 0.05) (Table 4). The independent variables of the three dimensions of learning attitude, engagement, and interpersonal relationships explained 71.3% of the model (Table 5), which provides a direction for future model improvement. PLAM should be adjusted more from the perspective of organizational style. Learning attitudes, participation, and interpersonal relationships had statistically significant effects on the evaluation of model effects (*P* < 0.05, as shown in Table 6).

**Table 4:** ANOVA of model effect evaluation models a

	Sum of squares	*df*	Mean square	*F*	Sig.
Return	32.025	4	8.006	59.434	.000^b^
Residual	12.124	90	0.135		
Total	44.149	94			

a. Dependent variable: model effect evaluation

b. Predictor variables: (constant), organization style, learning attitude, interpersonal relationship, participation

**Table 5:** Summary of model effect evaluation models ^b^

*R*	*R* ^2^	Adjust *R*^2^	Standard estimate error	Durbin-Watson
0.852^a^	0.725	0.713	0.36703	2.292

a. Predictor variables: (constant), organization style, learning attitude, interpersonal relationship, participation

b. Dependent variable: model effect evaluation

**Table 6:** Multiple linear regression analysis

Independent variable	Coefficient	Standard error	Standardization factor	*t*	Sig	VIF
(constant)	-0.062	0.373		-0.167	0.867	
Learning attitude	0.458***	0.065	0.533	7.064	0.000	1.868
Participation	0.305***	0.089	0.263	3.427	0.001	1.937
Interpersonal relationship	0.179 **	0.079	0.164	2.269	0.026	1.702
Organizational style	0.084	0.083	0.057	1.017	0.312	1.030

Note: *, **, *** indicate a significant correlation at the level of 10%, 5%, and 1%, respectively.

Learning attitude passed the 1% significance test and the coefficient was positive, indicating that learning attitude has a significant positive impact on the model evaluation effect. When students have better learning attitudes, the effect of the evaluation model is also better. Therefore, when promoting and applying the model, attention should be paid to cultivating students’ learning attitudes. Similarly, the degree of participation passed the 1% significance test, and the coefficient was positive, indicating that the degree of participation has a significant positive impact on the evaluation of the model. The more actively students participate in peer learning and mutual evaluation, the more feedback and gains they will derive from it, and the more obvious the improvement in their learning abilities will be. The interpersonal relationships within the group also have a significant positive impact on the evaluation effect of the model. More harmonious interpersonal relationships within the group equate to better student recognition of this model. Thus, we should ensure professional guidance for students’ cooperative learning with the aim of building a better emotional foundation. This is because in a good interpersonal atmosphere, students will be better able to participate in PLAM and are more likely to have the right attitude toward learning.

Finally, the organizational style did not show a significant impact in this study. This may be because of a lack of proficiency in the use of PLAM. First, we are not sufficiently trained to provide feedback to students, which means students themselves may not have been able to provide accurate feedback. Second, there were certain inappropriate aspects when allocating group members and dividing the workload for tasks, which led to some students’ ‘free-riding’ that may have affected other students’ enthusiasm for learning. Teachers should guide students to actively participate in PLAM in their learning. Therefore, improved implementation of PLAM will be a key focus for future research. We should improve the organization, for example, by making appropriate adjustments to group size, grouping methods, guiding group division of labor, and preventing ‘free-riding’ behavior. We have also made adjustments based on these aspects—the results of the follow-up survey are given in the Discussion section.

### One-sample ***t***-test

In this analysis, a one-sample *t*-test was used to determine the difference between the mean value of the sample’s evaluation of the model effect and the mean value of the scale (3.0). The results in Table S10 show that the mean value of the effect evaluation of the model was 3.973 ± 0.685, and the difference between the mean value of the effect evaluation and the mean value of the scale was 0.973 (95% confidence interval is 0.833–1.112). Results indicate a significant difference between the mean value of the model effect evaluation and the mean value of the scale, showing that in students’ subjective perceptions, PLAM can improve all aspects of their learning compared with when PLAM is not implemented.

### Word frequency analysis

**Fig. 2:**
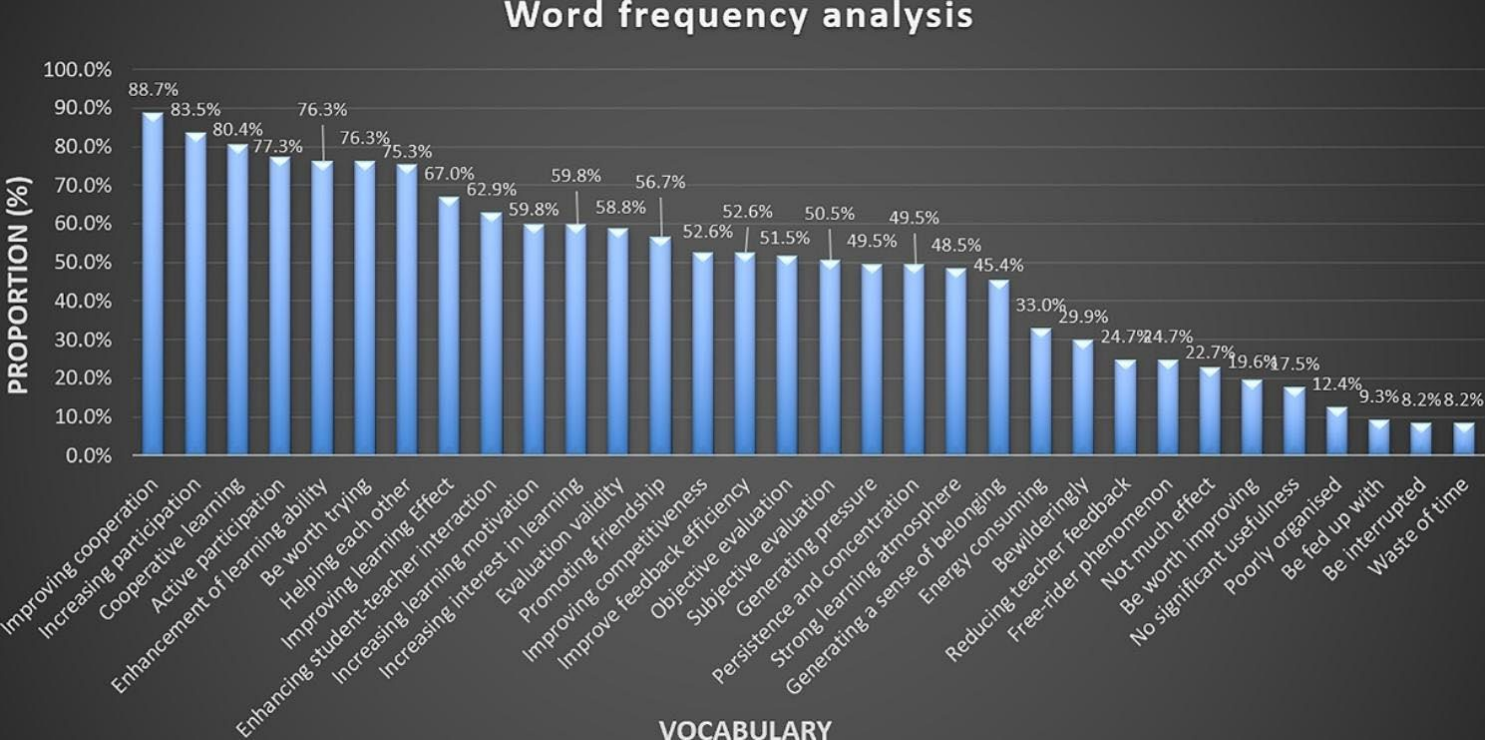
Word frequency analysis statistics chart Note: The horizontal axis indicates the 32 vocabulary items. The vertical coordinate shows the number of times the word/phrase was selected, and the data labels indicate the percentage of students who selected this option.

Word frequency analysis (WFA) is a statistical analysis of the number of occurrences of important words in text data, and an important tool in text mining. The basic principle is to identify hotspots and their trends through changes in the frequency of occurrence of words. To investigate student perceptions of PLAM that were more appropriate to our teaching practice in this instance, 32 descriptive words, both positive and negative, were selected from the literature related to peer learning and peer-to-peer assessment. In a follow-up supplementary survey, students were asked to indicate their innermost feelings by selecting the 10 words that best represented their feelings, irrespective of right or wrong. The results of the word frequency analysis are shown in Fig. 2. “Improving cooperation”, “Increasing participation”, “Cooperative learning”, “Active participation”, “Enhancement of learning ability”, “Be worth trying”, “Helping each other”, “Enhancing student-teacher interaction”, “Enhancing student-teacher interaction”, and “Increasing learning motivation” had the highest frequency.

While the greatest advantage of peer assessment was that it increased the effectiveness of feedback, PLAM also clearly enhanced learning collaboration and increased student participation in group learning. The terms “Enhancing student-teacher interaction” and “Increasing learning motivation” were also chosen with high frequency, suggesting it was the positive effects of PLAM that improved students’ learning ability and interest.

However, we also saw that student perceptions of “Improve feedback efficiency” were low, and there was a lack of agreement on the objective or subjective nature of the assessment, reflecting areas of PLAM that still require improvement, particularly with respect to training and the implementation of feedback. It is also possible that our questionnaire focused on the results of learning but ignored the learning process, because the result of improving feedback efficiency is increased learning effectiveness and interest, meaning that the students may have internalized the idea of “improving feedback efficiency” to such an extent that they did not feel it was necessary to select it.

### Supplementary survey

Based on the results of the first study, we adjusted the number of group members (reduced to 5–7) and provided guidance on group division of labor so that each student was given a specific task to prevent ‘free-riding.’ We administered the same questionnaire survey to two classes of students (94 in total) who took ‘Medicinal Chemistry of Natural Products’ in the follow-up and found the same trends. The results of the correlation and regression analyses are shown in Tables S11–14, and it is noteworthy that, after adjustment, all four dimensions were significantly correlated at the 99% level, indicating that the four dimensions interact with each other and that group size impacts the effectiveness of PLAM implementation. It also shows that our adjusted PLAM is more practical and scientific, and that 5–7 is a more appropriate group size. The supplemental survey (included in the Supplementary Information) also showed that 86% of students believe group size should not exceed 6 students.

The one-sample *t*-test (see Table S15 of the Supporting Information) showed that the difference between the mean ratings of the model’s effectiveness and the mean of the scale (3.0) indicated that, from students’ subjective perceptions, PLAM improves several aspects of student learning compared to when PLAM was not implemented – consistent with the conclusions of our initial investigation.

PLAM was popular among students, and only 17% noted that the group inter-assessment made them nervous. Almost half (48%) thought that the content of the group work itself took more time than usual, which suggests a need to adjust the workload. The supplementary survey results showed that most students (88.3%) chose a student with good organizational and coordination skills as the group leader. Thus, whether the training and selection of the group leader affects PLAM is also a factor worth exploring. Almost half the students (49%) sought to work with students who are well organized, suggesting that organizational and coordination skills are key abilities to develop in university education.

Regarding the role of the teacher in PLAM, 42.5% of the students thought that it was to organize the teaching and learning, 34% considered it was to review knowledge and give feedback, and 23.4% thought it was to guide the process of evaluation. This is generally consistent with our view that the teacher’s role in PLAM is to guide students in giving evaluations and to provide authoritative comments on the final results of group work to correct errors in group learning. However, the results of the regression analysis show that the independent variables of the four dimensions of learning attitude, engagement, interpersonal relationships, and organizational style explain 56.4% (Table S12) of the model. This suggests that PLAM needs further improvement, and limitations of the study and suggestions for addressing these in future research are outlined in the following paragraphs.

## Discussion

According to the 2020 statistics of the Ministry of Education of the People’s Republic of China (http://m.moe.gov.cn/jyb_xxgk/xxgk/neirong/tongji/gongbao/), enrolment figures in ordinary colleges and universities totaled 9,674,518, an increase of 5.74% from the previous year. There were 32,852,948 students in school, an increase of 8.37% from the previous year. However, there were 2,668,700 teaching staff in general higher education institutions, an increase of 3.97% from the previous year, and 1,833,000 full-time teachers, an increase of 5.34% over the previous year. The increase in teaching staff lags behind the growth in the number of students, and the student-teacher ratio increased to 18.37:1, almost reaching warning levels. As a result, the effectiveness of teachers’ assessment of students’ learning processes is bound to decrease, and appropriate teaching methods are necessary to improve the efficiency of feedback.

Scholars have shared a large number of successful applications of peer assessment. Results show that peer assessment can improve student performance and be applied to all types of students [46–49]. However, there are still few examples of the application of this teaching method in the education of Chinese university students, and a single peer assessment cannot solve the problems of delayed feedback and excessive class size in Chinese university education.

Our study suggests the PLAM we adopted improves the efficiency of student feedback, promotes collaborative learning, and increases enthusiasm and ability to learn. PLAM can be widely applied to different students because there are no significant differences in students’ evaluations of the model in terms of gender, grades, whether they have participated in peer assessment, or learning styles. The role of teachers in practical teaching requires increased guidance for lower-achieving students and helping all students actively participate in PLAM. Teachers need to develop a sense of cooperation among students, which will facilitate the implementation of PLAM. PLAM is more suitable for knowledge expansion learning and comprehensive practical learning. These type of curricula need to be tracked dynamically for the entire course duration, but teachers typically do not have sufficient time or energy to manage that process. PLAM is suitable for further extension in medical or pharmacy courses with similar characteristics to the course described in this paper.

We consider that PLAM can be further improved in three ways:

First, the experiment adopted a free grouping strategy considered humanistic-affective, with interpersonal relationships, academic performance and learning interest of group members not controlled by the teacher. Students may be more inclined to seek out classmates with whom they have a good relationship for collaborative learning. We do not know whether students will give higher marks to this close group of classmates or lower marks to their less well-connected classmates when assessing each other within the group. In our questionnaire results, interpersonal relationships had a significant effect on the evaluation of the effectiveness of PLAM, but our research in this area is insufficiently detailed. The question of how to achieve the optimal strategy regarding grouping methods, group size, members’ grades, and friendship requires further investigation. At the same time, we need to provide more guidance to students to reduce the negative impact of interpersonal relationships on learning and assessment.

Second, the evaluation of teachers’ perspectives could also be added to future questionnaires. Our initial intention was to reduce the pressure on teachers to provide student feedback. Thus, students were trained to take on some of the feedback tasks to make classroom feedback more effective. In PLAM, the teacher changes from being the sole evaluator to the secondary evaluator, and from the instructor to the supervisor. There is no denying that the teacher’s evaluation role in PLAM is diminished, but the teacher will give their authoritative evaluation at critical points to ensure the accuracy of knowledge. At other times, the giving of feedback is left to the students. The aim was to improve the effectiveness of feedback in the course – not to diminish the role of teachers in evaluation. PLAM accomplished the teaching tasks, and effective feedback from students was obtained. In the current student, we only collected research data from the perspective of students. In future investigations, teachers’ feedback should also be obtained.

Third, the details of PLAM require strengthening. Organizational style does not significantly influence the results. We trained and guided our students in PLAM practice by providing examples of teaching and assessment, and providing models and criteria for evaluation. However, vague and invalid evaluations were still received, reflecting that training was insufficient. Students with lower understanding or lacking evaluation experience struggle to give objective, accurate and meaningful evaluations. In future practice, proportion of grade composition, reasonable division of labor, and training of authentic ideas and critical thinking need continued investigation.

## Electronic supplementary material

Below is the link to the electronic supplementary material.

Supplementary Material 1

## Data Availability

The datasets analyzed during the current study are available from the corresponding author on reasonable request.

## Abbreviations

PLAM: Peer learning and assessment model
WFA: Word Frequency Analysis
