# Supplementary material for: Application of a peer learning and assessment model in an undergraduate pharmacy course
Source: BMC Med Educ. 2023 May 22;23:362. doi: 10.1186/s12909-023-04352-8 (PMC10204209; doi:10.1186/s12909-023-04352-8)
Supplement: Supplementary file 1 — Supplementary Material 1 [file 12909_2023_4352_MOESM1_ESM.docx]

Supplementary Information

**Application of a Peer Learning and Assessment Model in an Undergraduate Pharmacy Course**

Liyuan Yang, Yi Wang*

School of Medicine and Pharmacy, Ocean University of China; Laboratory for Marine Drugs and Bioproducts of Qingdao National Laboratory for Marine Science and Technology, Qingdao 266003, China.

[Table S1: Analysis of the differences of each dimension in the performance intervals 2](#_Toc132224349)

[Table S2: Analysis of the differences of each dimension in self-study methods 3](#_Toc132224350)

[Table S3: Analysis of the differences in the frequency of self-study in each dimension 4](#_Toc132224351)

[Table S4: Analysis of the differences of each dimension in gender 4](#_Toc132224352)

[Table S5: Analysis of the differences of each dimension in peer assessment experience 5](#_Toc132224353)

[Table S6: Analysis of differences in each dimension in the experience of serving as group leader 5](#_Toc132224354)

[Table S7: Analysis of the differences between different dimensions in learning approaches after class 6](#_Toc132224355)

[Table S8: Analysis of the differences of each dimension in learning styles 7](#_Toc132224356)

[Table S9: Correlation analysis between various dimensions 7](#_Toc132224357)

[Table S10 One-sample *t*-test 8](#_Toc132224358)

[Table S11 ANOVA of model effect evaluation models ^a^ 8](#_Toc132224359)

[Table S12 Summary of model effect evaluation models ^b^ 8](#_Toc132224360)

[Table S13 Multiple linear regression analysis 8](#_Toc132224361)

[Table S14: Correlation analysis between various dimensions 8](#_Toc132224362)

[Table S15 One-sample *t*-test 9](#_Toc132224363)

[**Test questionnaire** 9](#_Toc132224364)

[**Supplementary questionnaire** 11](#_Toc132224365)

| Table S1: Analysis of the differences of each dimension in the performance intervals | | | | | | | | | | | | |
| --- | --- | --- | --- | --- | --- | --- | --- | --- | --- | --- | --- | --- |
| Variable | Options | N | Mean | | Standard deviation | | *F* | | Sig | | Multiple comparisons after ANOVA | |
| Model effect evaluation | The top 20% | 18 | 4.00 | | 0.72 | | 1.129 | | 0.348 | | / | |
|  | 20%-40% | 21 | 4.12 | | 0.79 | |  |  |  |  |  |  |
|  | 40%-60% | 24 | 3.78 | | 0.56 | |  |  |  |  |  |  |
|  | 60%-80% | 21 | 4.11 | | 0.65 | |  |  |  |  |  |  |
|  | Last 20% | 11 | 3.79 | | 0.72 | |  |  |  |  |  |  |
| Learning attitude | The top 20% | 18 | 3.72 | | 0.75 | | 0.849 | | 0.498 | | / | |
|  | 20%-40% | 21 | 4.05 | | 1.02 | |  |  |  |  |  |  |
|  | 40%-60% | 24 | 3.67 | | 0.70 | |  |  |  |  |  |  |
|  | 60%-80% | 21 | 3.95 | | 0.67 | |  |  |  |  |  |  |
|  | Last 20% | 11 | 3.91 | | 0.83 | |  |  |  |  |  |  |
| Participation | The top 20% | 18 | 4.33 | | 0.45 | | 2.286 | | 0.066 | | 1>5,2>5,4>5 | |
|  | 20%-40% | 21 | 4.26 | | 0.63 | |  |  |  |  |  |  |
|  | 40%-60% | 24 | 4.01 | | 0.53 | |  |  |  |  |  |  |
|  | 60%-80% | 21 | 4.29 | | 0.57 | |  |  |  |  |  |  |
|  | Last 20% | 11 | 3.79 | | 0.74 | |  |  |  |  |  |  |
| Interpersonal relationship | The top 20% | 18 | 3.94 | | 0.64 | | 0.528 | | 0.715 | | / | |
|  | 20%-40% | 21 | 4.00 | | 0.55 | |  |  |  |  |  |  |
|  | 40%-60% | 24 | 3.88 | | 0.68 | |  |  |  |  |  |  |
|  | 60%-80% | 21 | 4.14 | | 0.65 | |  |  |  |  |  |  |
|  | Last 20% | 11 | 4.00 | | 0.63 | |  |  |  |  |  |  |
| Organizational style | The top 20% | 18 | 3.29 | | 0.38 | | 1.538 | | 0.198 | | / | |
|  | 20%-40% | 21 | 3.48 | | 0.47 | |  |  |  |  |  |  |
|  | 40%-60% | 24 | 3.58 | | 0.62 | |  |  |  |  |  |  |
|  | 60%-80% | 21 | 3.45 | | 0.33 | |  |  |  |  |  |  |
|  | Last 20% | 11 | 3.25 | | 0.33 | |  |  |  |  |  |  |
| Note: In multiple comparisons, 1 represents the top 20%, 2 represents 20%–40%, 3 represents 40%–60%, 4 represents 60%–80%, and 5 represents the bottom 20%. | | | | | | | | | | | | |
| Table S2: Analysis of the differences of each dimension in self-study methods | | | | | | | | | | | | |
| Variable | Options | N | | Mean | | Standard deviation | | *F* | | Sig | | Multiple comparisons after ANOVA |
| Model effect evaluation | Self-study independently | 67 | | 3.86 | | 0.67 | | 3.234 | | 0.044 | | 1<2 |
|  | Self-study together | 19 | | 4.25 | | 0.59 | |  |  |  |  |  |
|  | Basically no self-study | 9 | | 4.23 | | 0.81 | |  |  |  |  |  |
| Learning attitude | Self-study independently | 67 | | 3.76 | | 0.82 | | 1.562 | | 0.215 | | / |
|  | Self-study together | 19 | | 4.11 | | 0.66 | |  |  |  |  |  |
|  | Basically no self-study | 9 | | 4.00 | | 0.87 | |  |  |  |  |  |
| Participation | Self-study independently | 67 | | 4.06 | | 0.60 | | 3.372 | | 0.039 | | 1<2 |
|  | Self-study together | 19 | | 4.41 | | 0.50 | |  |  |  |  |  |
|  | Basically no self-study | 9 | | 4.36 | | 0.56 | |  |  |  |  |  |
| Interpersonal relationship | Self-study independently | 67 | | 3.91 | | 0.57 | | 1.917 | | 0.153 | | / |
|  | Self-study together | 19 | | 4.21 | | 0.63 | |  |  |  |  |  |
|  | Basically no self-study | 9 | | 4.11 | | 0.93 | |  |  |  |  |  |
| Organizational style | Self-study independently | 67 | | 3.45 | | 0.51 | | 0.294 | | 0.746 | | / |
|  | Self-study together | 19 | | 3.37 | | 0.36 | |  |  |  |  |  |
|  | Basically no self-study | 9 | | 3.49 | | 0.28 | |  |  |  |  |  |
| Note: In multiple comparisons, 1 represents independent self-study, 2 represents self-study together, 3 represents basically no self-study | | | | | | | | | | | | |

| Table S3: Analysis of the differences in the frequency of self-study in each dimension | | | | | | | |
| --- | --- | --- | --- | --- | --- | --- | --- |
| Variable | Options | N | Mean | Standard deviation | *F* | Sig | Multiple comparisons after ANOVA |
| Model effect evaluation | Once a week | 22 | 3.80 | 0.58 | 1.921 | 0.152 | / |
|  | 2-3 times a week | 35 | 4.14 | 0.61 |  |  |  |
|  | 4 times a week and above | 38 | 3.91 | 0.78 |  |  |  |
| Learning attitude | Once a week | 22 | 3.73 | 0.63 | 3.131 | 0.048 | 2>3 |
|  | 2-3 times a week | 35 | 4.11 | 0.80 |  |  |  |
|  | 4 times a week and above | 38 | 3.68 | 0.84 |  |  |  |
| Participation | Once a week | 22 | 3.99 | 0.51 | 1.217 | 0.301 | / |
|  | 2-3 times a week | 35 | 4.24 | 0.60 |  |  |  |
|  | 4 times a week and above | 38 | 4.19 | 0.62 |  |  |  |
| Interpersonal relationship | Once a week | 22 | 3.82 | 0.59 | 2.605 | 0.079 | / |
|  | 2-3 times a week | 35 | 4.17 | 0.62 |  |  |  |
|  | 4 times a week and above | 38 | 3.92 | 0.63 |  |  |  |
| Organizational style | Once a week | 22 | 3.42 | 0.52 | 0.186 | 0.830 | / |
|  | 2-3 times a week | 35 | 3.48 | 0.47 |  |  |  |
|  | 4 times a week and above | 38 | 3.41 | 0.44 |  |  |  |

Note: In multiple comparisons, 1 means once a week, 2 means self-study 2-3 times a week, 3 means self-study 4 times a week or more

| Table S4: Analysis of the differences of each dimension in gender | | | | | |
| --- | --- | --- | --- | --- | --- |
| Variable | Options | N | Mean | *t* | Sig. (two-sided) |
| Model effect evaluation | Male | 29 | 3.97 | -0.067 | 0.947 |
|  | Female | 66 | 3.98 |  |  |
| Learning attitude | Male | 29 | 3.93 | 0.632 | 0.529 |
|  | Female | 66 | 3.82 |  |  |
| Participation | Male | 29 | 4.09 | -0.725 | 0.470 |
|  | Female | 66 | 4.19 |  |  |
| Interpersonal relationship | Male | 29 | 4.03 | 0.462 | 0.645 |
|  | Female | 66 | 3.97 |  |  |
| Organizational style | Male | 29 | 3.45 | 0.203 | 0.839 |
|  | Female | 66 | 3.43 |  |  |

| Table S5: Analysis of the differences of each dimension in peer assessment experience | | | | | |
| --- | --- | --- | --- | --- | --- |
| Variable | Have you participated in peer assessment | N | Mean | *t* | Sig. (two-sided) |
| Model effect evaluation | Yes | 86 | 3.98 | 0.486 | 0.628 |
|  | No | 9 | 3.87 |  |  |
| Learning attitude | Yes | 86 | 3.87 | 0.733 | 0.466 |
|  | No | 9 | 3.67 |  |  |
| Participation | Yes | 86 | 4.19 | 1.281 | 0.203 |
|  | No | 9 | 3.92 |  |  |
| Interpersonal relationship | Yes | 86 | 3.98 | -0.609 | 0.544 |
|  | No | 9 | 4.11 |  |  |
| Organizational style | Yes | 86 | 3.44 | 0.027 | 0.978 |
|  | No | 9 | 3.43 |  |  |

| Table S6: Analysis of differences in each dimension in the experience of serving as group leader | | | | | |
| --- | --- | --- | --- | --- | --- |
| Variable | Whether to serve as team leader | N | Mean | *t* | Sig. (two-sided) |
| Model effect evaluation | Yes | 20 | 4.07 | 0.676 | 0.500 |
|  | No | 75 | 3.95 |  |  |
| Learning attitude | Yes | 20 | 3.85 | -0.016 | 0.987 |
|  | No | 75 | 3.85 |  |  |
| Participation | Yes | 20 | 4.37 | 1.801 | 0.075 |
|  | No | 75 | 4.10 |  |  |
| Interpersonal relationship | Yes | 20 | 3.90 | -0.716 | 0.476 |
|  | No | 75 | 4.01 |  |  |
| Organizational style | Yes | 20 | 3.40 | -0.354 | 0.724 |
|  | No | 75 | 3.45 |  |  |

| Table S7: Analysis of the differences between different dimensions in learning approaches after class | | | | | | |
| --- | --- | --- | --- | --- | --- | --- |
| Variable | Options | N | Mean | Standard deviation | *F* | Sig |
| Model effect evaluation | Tablet | 19 | 4.05 | 0.58 | 1.953 | 0.148 |
|  | Textbooks and handouts | 70 | 4.00 | 0.69 |  |  |
|  | Other | 6 | 3.45 | 0.81 |  |  |
| Learning attitude | Tablet | 19 | 3.95 | 0.62 | 2.484 | 0.089 |
|  | Textbooks and handouts | 70 | 3.89 | 0.81 |  |  |
|  | Other | 6 | 3.17 | 0.98 |  |  |
| Participation | Tablet | 19 | 4.14 | 0.64 | 0.546 | 0.581 |
|  | Textbooks and handouts | 70 | 4.19 | 0.59 |  |  |
|  | Other | 6 | 3.93 | 0.49 |  |  |
| Interpersonal relationship | Tablet | 19 | 4.05 | 0.52 | 0.279 | 0.757 |
|  | Textbooks and handouts | 70 | 3.99 | 0.65 |  |  |
|  | Other | 6 | 3.83 | 0.75 |  |  |
| Organizational style | Tablet | 19 | 3.37 | 0.38 | 0.510 | 0.602 |
|  | Textbooks and handouts | 70 | 3.44 | 0.45 |  |  |
|  | Other | 6 | 3.59 | 0.87 |  |  |

| Table S8: Analysis of the differences of each dimension in learning styles | | | | | | |
| --- | --- | --- | --- | --- | --- | --- |
| Variable | Options | N | Mean | Standard deviation | *F* | Sig |
| Model effect evaluation | Divergent | 35 | 3.92 | 0.62 | 0.670 | 0.573 |
|  | Assimilator | 30 | 4.07 | 0.74 |  |  |
|  | Gatherer | 7 | 4.17 | 0.60 |  |  |
|  | Adaptor | 23 | 3.87 | 0.74 |  |  |
| Learning attitude | Divergent | 35 | 3.86 | 0.65 | 0.366 | 0.778 |
|  | Assimilator | 30 | 3.83 | 0.83 |  |  |
|  | Gatherer | 7 | 4.14 | 1.07 |  |  |
|  | Adaptor | 23 | 3.78 | 0.90 |  |  |
| Participation | Divergent | 35 | 4.27 | 0.51 | 1.831 | 0.147 |
|  | Assimilator | 30 | 4.20 | 0.57 |  |  |
|  | Gatherer | 7 | 4.27 | 0.53 |  |  |
|  | Adaptor | 23 | 3.92 | 0.71 |  |  |
| Interpersonal relationship | Divergent | 35 | 3.91 | 0.61 | 0.686 | 0.563 |
|  | Assimilator | 30 | 4.00 | 0.64 |  |  |
|  | Gatherer | 7 | 4.29 | 0.49 |  |  |
|  | Adaptor | 23 | 4.00 | 0.67 |  |  |
| Organizational style | Divergent | 35 | 3.54 | 0.47 | 2.087 | 0.107 |
|  | Assimilator | 30 | 3.47 | 0.54 |  |  |
|  | Gatherer | 7 | 3.46 | 0.37 |  |  |
|  | Adaptor | 23 | 3.24 | 0.33 |  |  |

| Table S9: Correlation analysis between various dimensions | | | | | | |
| --- | --- | --- | --- | --- | --- | --- |
| Variable | Correlation | Model effect evaluation | Learning attitude | Participation | Interpersonal relationship | Organizational style |
| Model effect evaluation | Pearson Correlation | 1 |  |  |  |  |
| Learning attitude | Pearson Correlation | .799** | 1 |  |  |  |
| Participation | Pearson Correlation | .708** | .636** | 1 |  |  |
| Interpersonal relationship | Pearson Correlation | .630** | .570** | .587** | 1 |  |
| Organizational style | Pearson Correlation | .168 | .089 | .154 | .141 | 1 |
| **. Significantly correlated at the .01 level (two-sided) | | | | | | |

| Table S10 One-sample *t*-test | | | | | | | | | |
| --- | --- | --- | --- | --- | --- | --- | --- | --- | --- |
| Variable | Mean | Standard deviation | Test value = 3 | | | | | | |
|  |  |  | *t* | *df* | Sig.  (two-sided) | Mean difference | 95% confidence interval of the difference | | |
|  |  |  |  |  |  |  | Lower limit | Upper limit | |
| Model effect evaluation | 3.973 | 0.685 | 13.833 | 94 | 0.000 | 0.973 | 0.833 | | 1.112 |

| Table S11 ANOVA of model effect evaluation models ^a^ | | | | | |
| --- | --- | --- | --- | --- | --- |
|  | Sum of squares | *df* | Mean square | *F* | Sig. |
| Return | 20.675 | 4 | 5.169 | 31.044 | 0.000^b^ |
| Residual | 14.818 | 89 | 0.166 |  |  |
| Total | 35.493 | 93 |  |  |  |
| a. Dependent variable: model effect evaluation | | | | | |
| b. Predictor variables: (constant), organization style, learning attitude, interpersonal relationship, participation | | | | | |

| Table S12 Summary of model effect evaluation models ^b^ | | | | |
| --- | --- | --- | --- | --- |
| *R* | *R^2^* | Adjust *R^2^* | Standard estimate error | Durbin-Watson |
| 0.763^a^ | 0.583 | 0.564 | 0.4080 | 1.684 |
| a. Predictor variables: (constant), organization style, learning attitude, interpersonal relationship, participation | | | | |
| b. Dependent variable: model effect evaluation | | | | |

| Table S13 Multiple linear regression analysis | | | | | | |
| --- | --- | --- | --- | --- | --- | --- |
| Independent variable | Coefficient | Standard error | Standardization factor | t | Sig | VIF |
| (constant) | -0.243 | 0.287 |  | -0.848 | 0.399 | -0.243 |
| Learning attitude | 0.373 | 0.084 | 0.409 | 4.462 | 0.000 | 0.373 |
| Participation | -0.026 | 0.118 | -0.021 | -0.221 | 0.826 | -0.026 |
| Interpersonal relationship | 0.510 | 0.092 | 0.448 | 5.520 | 0.000 | 0.510 |
| Organizational style | 0.212 | 0.157 | 0.121 | 1.351 | 0.180 | 0.212 |
|  | | | | | | |

| Table S14: Correlation analysis between various dimensions | | | | | | |
| --- | --- | --- | --- | --- | --- | --- |
| Variable | Correlation | Model effect evaluation | Learning attitude | Participation | Interpersonal relationship | Organizational style |
| Model effect evaluation | Pearson Correlation | 1 |  |  |  |  |
| Learning attitude | Pearson Correlation | .595** | 1 |  |  |  |
| Participation | Pearson Correlation | .474** | .650** | 1 |  |  |
| Organizational style | Pearson Correlation | .525** | .447** | .526** | 1 |  |
| Interpersonal relationship | Pearson Correlation | .636** | .326** | .370** | .521** | 1 |
| **. Significantly correlated at the .01 level (two-sided) | | | | | | |

| Table S15 One-sample *t*-test | | | | | | | | | |
| --- | --- | --- | --- | --- | --- | --- | --- | --- | --- |
| Variable | Mean | Standard deviation | Test value = 3 | | | | | | |
|  |  |  | *t* | *df* | Sig.  (two-sided) | Mean difference | 95% confidence interval of the difference | | |
|  |  |  |  |  |  |  | Lower limit | Upper limit | |
| Model effect evaluation | 3.946 | 0.618 | 14.842 | 93 | 0.000 | 0.946 | 0.819 | | 1.072 |

#

# **Test questionnaire**

Dear students, please answer the following questions according to your actual situation and true feelings. There are no right or wrong answers. The questionnaire data will only be used for educational research.

| **Basic Information** | | | | | |
| --- | --- | --- | --- | --- | --- |
| 1. Your name:  2. Your Student ID:  3. Your grade and major: | | | | | |
| **Interviewees’ Basic Personal Characteristics** | | | | | |
| 1. Gender：  A. Male B. Female  2. GPA ranking during undergraduate studies:  A. Top 20% B. 20%–40% C. 40%–60% D. 60%–80% E. Bottom 20%  3. Learning style：  (1) Self-study method：  A. Self-study independently B. Self-study together C. Basically no self-study  (2) Self-study frequency：  A. Once a week B. 2–3 times a week C. 4 times a week or more often  (3) Self-study approach：  A. Tablet B. Textbooks and handouts C. Other  4. Peer assessment experience  （1）Have you participated in peer assessment? A. Yes B. No  （2）If so, have you served as a team leader? A. Yes B. No  5. Your learning style belongs to the following category:  A. Divergent: Tends to use concrete experience and reflective observation, and to be good at observing others and collecting information. Tends to have a wide range of interests, a strong imagination, and a good understanding of things.  B. Assimilator: Tends to use abstract concepts and reflective observation to learn and to be good at processing large amounts of information. Tends to have a strong inductive reasoning ability and to be good at establishing a theoretical framework and forming logical and accurate ideas.  C. Gatherer: Tends to actively experiment and learn abstract concepts and to be good at applying theory to practice. Tends to have strong problem-solving abilities and to be good at applying knowledge to practice.  D. Adaptor: Tends to experiment actively, to rely on concrete experience, and to be good at gaining experience from practice. Tends to have a strong ability to deal with crises and look for opportunities. Often deals with problems by using intuition and trial and error, and tends to rely on information provided by others rather than personal analysis. | | | | | |
| 1~5 From “strongly agree” to “strongly disagree,” please tick “√” in the corresponding box (please answer the questions according to your real feelings) | Strongly agree | Agree | Neither agree nor disagree | Disagree | Strongly disagree |
| **Learning attitude** | | | | | |
| 1. I think PLAM is very important |  |  |  |  |  |
| 2. I think PLAM is helpful for studying this course |  |  |  |  |  |
| 3. I agree that teachers should adopt PLAM in teaching |  |  |  |  |  |
| 4. I look forward to encountering my classmates’ thinking and expanding my own thinking in group study |  |  |  |  |  |
| 5. Feedback from group members is more effective than feedback from teachers |  |  |  |  |  |
| **Participation** | | | | | |
| 1. I can take peer review seriously |  |  |  |  |  |
| 2. Group evaluation can improve the quality of students’ work |  |  |  |  |  |
| 3. I take an active attitude toward group cooperative learning |  |  |  |  |  |
| 4. I can take an active part in every group discussion |  |  |  |  |  |
| 5. I can actively answer the questions of the group members |  |  |  |  |  |
| 6. When I do not understand, I will actively ask team members for help |  |  |  |  |  |
| 7. I often share information or experiences with group members |  |  |  |  |  |
| **Interpersonal relationships** | | | | | |
| 1. I think peer review would hurt my friendships |  |  |  |  |  |
| 2. I think peer review will enhance my friendships |  |  |  |  |  |
| 3. I rate people with whom I have a good relationship higher |  |  |  |  |  |
| 4. The members of my study group get along well with each other |  |  |  |  |  |
| 5. The members of my study group help each other and cooperate well |  |  |  |  |  |
| **Organizational style** | | | | | |
| 1. The group comments make me nervous |  |  |  |  |  |
| 2. The group work takes me a lot of time |  |  |  |  |  |
| 3. I don’t know how to give advice to team members and other team results |  |  |  |  |  |
| 4. The evaluations of team members are able to truthfully reflect the quality of my work |  |  |  |  |  |
| 5. A brief comment would have helped me more |  |  |  |  |  |
| 6. The group evaluations make me feel competitive |  |  |  |  |  |
| 7. The group evaluations make me feel cooperative |  |  |  |  |  |
| 8. My group has a clear division of tasks |  |  |  |  |  |
| 9. Members of my group engage in some free-riding behavior |  |  |  |  |  |
| **Effect evaluation model** | | | | | |
| 1. Group evaluations and inter-group evaluations help me identify my shortcomings |  |  |  |  |  |
| 2. PLAM can improve my independent thinking ability |  |  |  |  |  |
| 3. PLAM enables me to better understand my coursework and gain knowledge |  |  |  |  |  |
| 4. PLAM can improve my language expression ability |  |  |  |  |  |
| 5. PLAM can improve my independent learning ability |  |  |  |  |  |
| 6. PLAM can improve my text-reading ability |  |  |  |  |  |
| 7. PLAM can improve my communication skills |  |  |  |  |  |
| 8. PLAM can improve my creative ability |  |  |  |  |  |
| 9. PLAM can improve my interest in Medicinal Chemistry of Natural Products |  |  |  |  |  |
| 10. PLAM can improve my interest in pharmacy |  |  |  |  |  |

**Note：The test questionnaire was administered in Chinese when collecting the data.**

# **Supplementary Questionnaire**

| **Basic Information** | | | | | |
| --- | --- | --- | --- | --- | --- |
| 1. Your name:  2. Your Student ID:  3. Your grade and major: | | | | | |
| **Additional Questions** | | | | | |
| 1. The way your group is grouped is:  A. Free Teaming B. Teacher grouping  2. What do you think is a better way to form groups?  A. Free Teaming B. Teacher grouping C. Doesn’t matter  3. Which of the following types of students would you prefer to elect as group leaders?  A. Students with good academic performance B. Students who have a good relationship with yourself  C. Students with good organization and coordination skills D. Students who are active in class  4. If you are free to form a team, which of the following types of students would you prefer to work with?  A. Students with good academic performance B. Students who have a good relationship with yourself  C. Students with good organization and coordination skills D. Students who are active in class  5. What do you think is the role of teachers in the peer learning assessment model?  A. Guiding how to evaluate B. Organize teaching and learning properly  C. Point out the knowledge and give feedback D. Not much of a role | | | | | |
| 1~5 From “very difficult” to “very easy,” please tick “√” in the corresponding box (please answer the questions according to your real feelings) | Very difficult | More difficult | Fair | Easier | Very easy |
| How difficult do you think this group work is? |  |  |  |  |  |
